# Quercetin-4′-*O*-β-D-glucopyranoside (QODG) Inhibits Angiogenesis by Suppressing VEGFR2-Mediated Signaling in Zebrafish and Endothelial Cells

**DOI:** 10.1371/journal.pone.0031708

**Published:** 2012-02-13

**Authors:** Chen Lin, Menghua Wu, Jianyong Dong

**Affiliations:** Pharmacy School, Wenzhou Medical College, Wenzhou, Zhejiang Province, People's Republic of China; The University of Birmingham, United Kingdom

## Abstract

**Background:**

Angiogenesis plays an important role in many physiological and pathological processes. Identification of small molecules that block angiogenesis and are safe and affordable has been a challenge in drug development. *Hypericum attenuatum* Choisy is a Chinese herb medicine commonly used for treating hemorrhagic diseases. The present study investigates the anti-angiogenic effects of quercetin-4′-*O*-β-D-glucopyranoside (QODG), a flavonoid isolated from *Hypericum attenuatum* Choisy, *in vivo* and *in vitro*, and clarifies the underlying mechanism of the activity.

**Methodology/Principal Findings:**

*Tg(fli1:EGFP)* transgenic zebrafish embryos were treated with different concentrations of quercetin-4′-*O*-β-D-glucopyranoside (QODG) (20, 60, 180 µM) from 6 hours post fertilisation (hpf) to 72 hpf, and adult zebrafish were allowed to recover in different concentrations of QODG (20, 60, 180 µM) for 7 days post amputation (dpa) prior morphological observation and angiogenesis phenotypes assessment. Human umbilical vein endothelial cells (HUVECs) were treated with or without VEGF and different concentrations of QODG (5, 20, 60, 180 µM), then tested for cell viability, cell migration, tube formation and apoptosis. The role of VEGFR2-mediated signaling pathway in QODG-inhibited angiogenesis was evaluated using quantitative real-time PCR (qRT-PCR) and Western blotting.

**Conclusion/Significance:**

Quercetin-4′-*O*-β-D-glucopyranoside (QODG) was shown to inhibit angiogenesis in human umbilical vein endothelial cells (HUVECs) *in vitro* and zebrafish *in vivo* via suppressing VEGF-induced phosphorylation of VEGFR2. Our results further indicate that QODG inhibits angiogenesis via inhibition of VEGFR2-mediated signaling with the involvement of some key kinases such as c-Src, FAK, ERK, AKT, mTOR and S6K and induction of apoptosis. Together, this study reveals, for the first time, that QODG acts as a potent VEGFR2 kinase inhibitor, and exerts the anti-angiogenic activity at least in part through VEGFR2-mediated signaling pathway.

## Introduction

Angiogenesis, the formation of new blood vessels by sprouting from pre-existing endothelium, is a significant component of a wide variety of biological processes, including embryonic vascular development, differentiation, wound healing and organ regeneration [1], [2], and pathological processes, including tumor progression [1]–[13], infection [2], ischaemic and inflammatory diseases [2], [11], and several other disorders, for example, arthritis, psoriasis, atherosclerosis, metastasis disorder [1], immune disorders [2], age-related macular degeneration [6], [9], and diabetic retinopathy [1], [9], [14]. Angiogenesis is tightly regulated by an intricate balance between the angiogenic and anti-angiogenic factors [13]. Of the numerous growth factors and cytokines that have been shown angiogenic effects, vascular endothelial growth factor (VEGF), a glycoprotein that has mitogenic activity on vascular endothelial cells, is one of the most critical and specific angiogenic factors regulating normal physiological and pathological neovascularization such as tumor angiogenesis [6], [15]–[17]. VEGF exerts its biological actions by binding to its two receptor tyrosine kinases expressed on endothelial cells, namely, VEGFR1 (Flt-1) and VEGFR2 (KDR/Flk-1). VEGFR1 is poorly autophosphorylated in response to VEGF in endothelial cells and is weakly involved in transducing the VEGF angiogenic signals. The evidences supports the concept that VEGFR1 might act as a decoy receptor rather than as a signal-transducing molecule [18], [19], whereas ligand-induced homodimerization of VEGFR2 leads to a strong autophosphorylation of several tyrosine residues of VEGFR2 [20]. VEGFR2 is essential for the morphogenesis of vascular endothelium and is the primary receptor mediating the angiogenic activity of VEGF through distinct signal transduction pathways that regulate endothelial cell proliferation, migration, differentiation and tube formation [15], [21], [22]. The VEGFR2 signaling pathway is a promising target of angiogenesis, because it is a common pathway for tumor-induced angiogenesis [23].

Interestingly, activation of VEGFR2 by VEGF results in the activation of diverse signaling molecules, such as Src family kinase [24], focal adhesion kinase (FAK) [25], [26], extracellular signal-related kinase (ERK) [26], [27], AKT/protein kinase B (PKB) [26], mammalian target of rapamycin (mTOR) [28] and ribosomal protein S6 kinase (p70S6K) [29], which promote the growth, migration, differentiation and survival of endothelial cells in pre-existing vasculature.

VEGF is viewed as an attractive therapeutic target for the development of novel anticancer agents [6]. There are several angiogenesis inhibitors in phase I or phase II clinical trials, including antibodies aimed at VEGF or VEGFRs [30], [31], soluble decoy receptors that sequester ligands [32] and small molecule inhibitors that inhibit kinase activity [33]. Three anti-angiogenic drugs, bevacizumab (Avastin®), sunitinib malate (Sutent®, SU11248) and sorafenib (Nexavar®, BAY 43-9006), inhibiting VEGF signaling by either blocking VEGF ligands or VEGFRs, have been approved by the United States Food and Drug Administration for cancer treatment [34].

However, serious side effects, such as hypertension, bleeding and gastrointestinal perforation, have been associated with currently available anti-angiogenic agents, limiting their chronic use [33]. To exploit more efficient and safer agents for the treatment of angiogenesis-related diseases such as cancer, a large number of scholars have been actively pursuing small molecule therapeutic strategies targeted at VEGFR2-mediated signal transduction pathway [35], [36].

There has, consequently, been a renewed interest in identifying natural products, such as certain Chinese herbal medicines, which contain a variety of anti-angiogenic compounds, and are given the advantage of proven safety for human use. Current knowledge regarding the anti-angiogenic potential of natural products has demonstrated that flavonoid constituents in Gingko biloba and Genistein (a soy isoflavone) are considered to exert potent anti-angiogenic property [37], [38]. Another important compound, Hyperforin, a phloroglucinol derivative found in St. John's wort (SJW, *Hypericum perforatum*) related mainly to its anti-depressant effects, has been reported recently to interfere with key events in angiogenesis, and be a promising drug for the treatment of angiogenesis-related diseases [39]. Hyperforin can induce apoptosis in tumor cells, inhibit cancer invasion and metastasis [39], [40], inhibit angiogenesis *in vitro* in bovine aortic endothelial cells as well as *in vivo* in the chorioallantoic membrane [39] and Wistar rats [41].

Because of the potential of anti-angiogenesis both found in flavonoids and *Hypericum*, we researched the effects of flavonoids in *Hypericum attenuatum* Choisy on angiogenesis, to obtain new drugs for angiogenesis treatment. *Hypericum attenuatum* Choisy is a Chineses native herbal medicine, which is rich in flavonids, and often used either as a single herb or in combination with other Chinese herbal medicines as formula for treating haemoptysis, haematemesis, metrorrhagia, traumatichemorrhage, rheumatoid arthralgia, neuralgia, injury, blurred vision and pyogenicinfection, among others.

**Figure 1:**
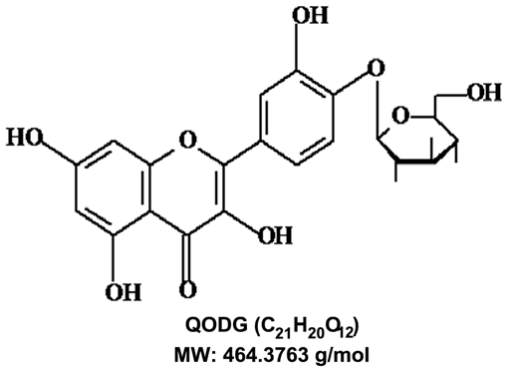
Chemical structure of quercetin-4′-O-β-D-glucopyranoside (QODG). Quercetin-4′-O-β-D-glucopyranoside (QODG) has a molecular formula C_21_H_20_O_12_ with a molecular weight of 464.3763 g/mol.

The major bioactive constituents of *Hypericum attenuatum* Choisy are flavonoids, including quercetin, quercitrin, isoquercitrin, rutin [42], quercetin-4′-*O*-β-D-glucopyranoside. Among these flavonoids, we have found quercetin-4′-*O*-β-D-glucopyranoside (QODG), a bioactive flavonoid with a molecular weight of 464.3763 g/mol (Fig. 1), is a candidate with the fullest potential to develop as a small-molecule anti-angiogenic agent, due to the anti-angiogenic activity showed in zebrafish (*Danio rerio*), a useful model for high-throughput screening drugs and compounds which have effects on the vasculature [43], [44].

In recent assays for angiogenesis, the zebrafish model is practical and efficient in screening drug for pro-angiogenesis or anti-angiogenesis [45]–[49]. The zebrafish embryo is an excellent model to use for investigating vascular development. The transparency of zebrafish embryos allows for convenient observation during development while *Tg(fli1:EGFP)* transgenic line expressing fluorescent tags in endothelial cells facilitates the study of developing blood vessels [50], [51]. Zebrafish vasculogenesis and angiogenesis are two distinct vascular processes and occur at different phases of vascular development. During zebrafish development, the *de novo* formation of the dorsal aorta and the posterior cardinal vein of the tail occurs via the fusion of angioblast precursor cells and is considered vasculogenesis. The subsequent sprouting and extension of the intersegmental vessels (ISVs) from the dorsal aorta is considered angiogenesis [52].

In addition, another crucial consideration is that many human diseases concerning angiogenesis occur during adulthood, for example, diabetic retinopathy. Although a biologically active chemical may act on the same protein(s), the physiological outcome in an embryo could be distinct from its effects in an adult owing to the different needs for target protein function. Thus, testing chemicals in an adult model organism should provide additional insights into their effects on biology. The adult zebrafish has also been used as a model for angiogenesis, in particular, zebrafish caudal fin regeneration provides a platform for evaluating anti-angiogenic therapy and discovering biologically active chemical compounds [53]. In zebrafish caudal fins, blood vessels play an important role in the patterning of bony rays [54]. The adult zebrafish is used as a new model system for studying receptor signaling and chemical biology as well [53].

Therefore, in our study we chose *Tg(fli1:EGFP)* transgenic zebrafish model (both zebrafish embryos and adult zebrafish) to examine the effects of quercetin-4′-*O*-β-D-glucopyranoside (QODG) on blood vessels and to examine the molecular mechanism for QODG-mediated inhibition of angiogensis. We found that QODG significantly inhibited angiogenesis in zebrafish, also strongly inhibited endothelial cell proliferation, migration and tube formation, as well as induced apoptosis in HUVECs. Furthermore, we revealed that QODG exerted its anti-angiogenic activity through the inhibition of VEGFR2 tyrosine kinase activity and VEGFR2-mediated signaling pathway.

## Results

### QODG inhibits angiogenesis in the zebrafish model

**Figure 2:**
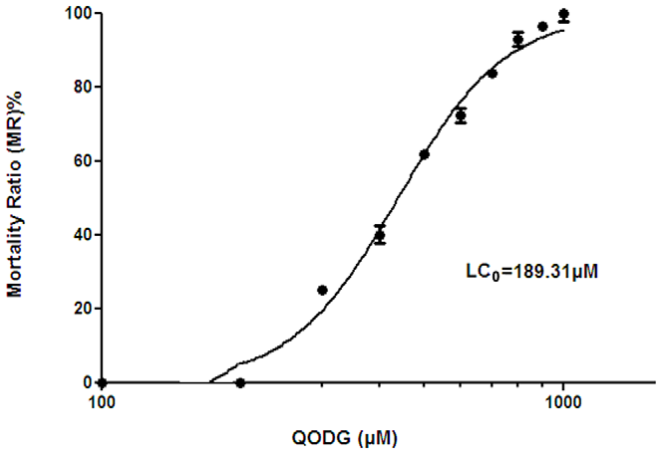
Toxic effects of QODG on zebrafish. *Tg(fli1:EGFP)* zebrafish embryos were treated with various concentrations of QODG (100, 200, 300, 400, 500, 600, 700, 800, 900, 1000 µM) from 6 hours post fertilization (hpf) to 72 hpf. During the period, the fish were observed for survival and morphology under an inverted microscope (at both 10×magnification and 100×magnification). Data were analyzed by statistical package SPSS 17.0 for non-linear regression from three independent experiments, and LC_0_ (i.e., LC_min_) was defined as the minimum lethal concentration that resulted in 0% mortality of zebrafish treated with QODG.

**Figure 3:**
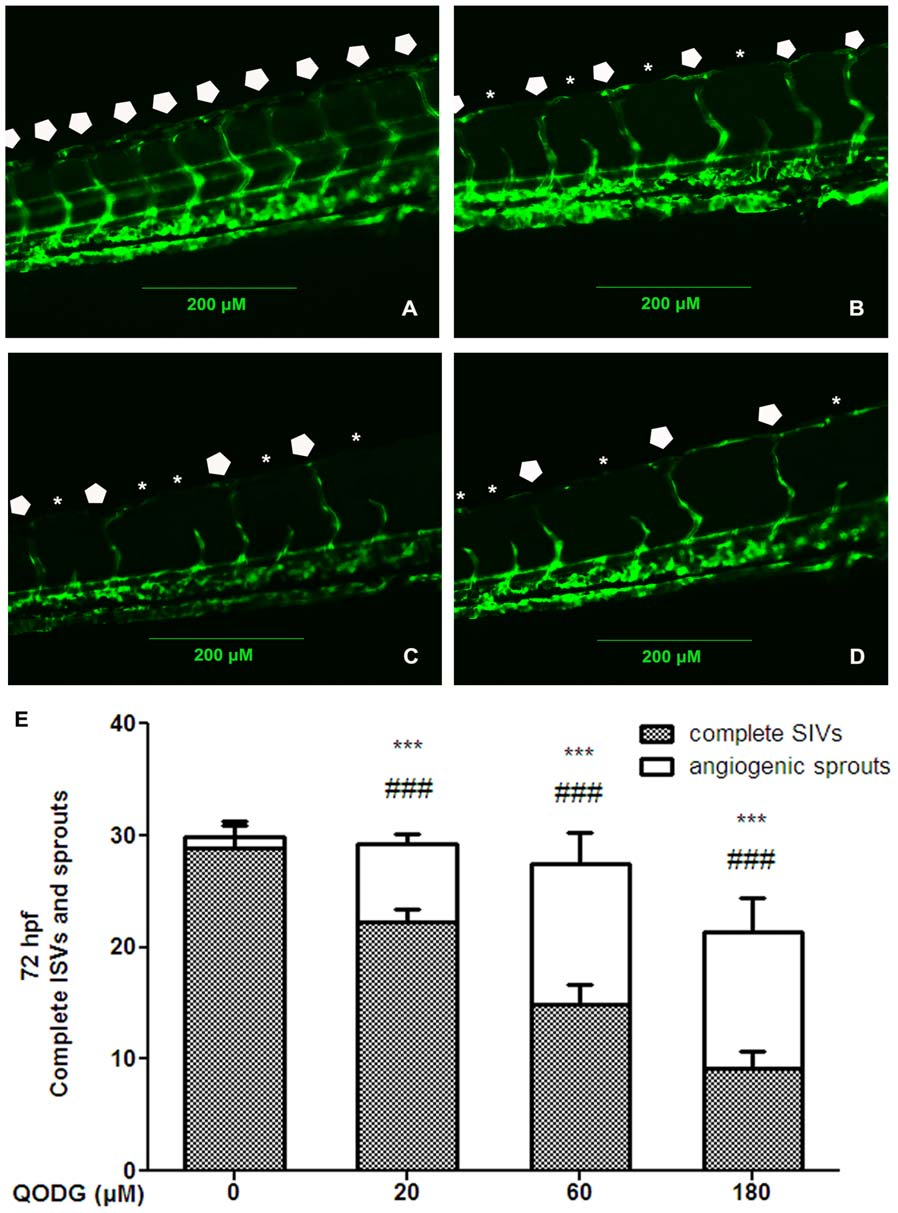
QODG inhibits blood vessel formation in ISVs of zebrafish embryos. (A) Vehicle control: *Tg(fli1:EGFP)* zebrafish embryos were treated with 0.1% DMSO from 6 hours post fertilization (hpf) to 72 hpf. All intersegmental vessels (ISVs) in the vehicle control group have fully extended to form the dorsal longitudinal anastomotic vessels (DLAVs) at 72 hpf. (B–D) QODG-treated groups: *Tg(fli1:EGFP)* zebrafish embryos were treated with various concentrations of QODG (20, 60, 180 µM) from 6 hpf to 72 hpf. Pentagons indicate the sites of complete ISVs in zebrafish embryos for all figures, and asterisks indicate the sites of angiogenic sprouts in zebrafish embryos for all figures. (E) Quantitative comparison of blood vessel formation in the vehicle control group and QODG-treated groups. Data are expressed as mean ± SD from three independent experiments. *, the number of complete ISVs in QODG-treated group compared with that in the vehicle control group; ***, P<0.001 vs. vehicle control. ^#^, the number of angiogenic sprouts in QODG-treated group compared with that in the vehicle control group; ^###^, P<0.001 vs. vehicle control. Scale bars, 200 µm.

To assess the anti-angiogenic property of QODG *in vivo*, we examined the inhibitory effects of QODG on zebrafish blood vessel development. However, in order to exclude the possibility that the anti-angiogenic effects generated by QODG were related to its toxicity *in vivo*, we firstly examined the toxic effects of QODG on zebrafish, and we found that the minimum lethal concentration (LC_min_, i.e., LC_0_) of QODG in this model was 189.31 µM (Fig. 2). When treated with 180 µM QODG, zebrafish were not shown malformed in the morphology, that is to say, 180 µM was approximately the maximum safe concentration in zebrafish. For this reason, 180 µM was designed as the highest concentration for further experiments in zebrafish. *Tg(fli1:EGFP)* transgenic zebrafish embryos were treated with DMSO (0.1%) or different concentrations of QODG (20, 60, 180 µM) from the shield stage (6 hpf) to 72 hpf (hours post fertilisation). When zebrafish were analyzed at 72 hpf, a time point where all intersegmental vessels (ISVs) in the vehicle control group treated with 0.1% DMSO from 6 hpf to 72 hpf have fully extended to form the dorsal longitudinal anastomotic vessels (DLAVs), QODG-treated groups were found to result in significant reductions in the number of complete ISVs and angiogenic sprouts compared with the vehicle control group, with a greatest reduction in embryos treated with 180 µM QODG (Fig. 3, p<0.001). These results demonstrate that QODG serves as an inhibitor of ISVs angiogenesis in zebrafish embryos.

**Figure 4:**
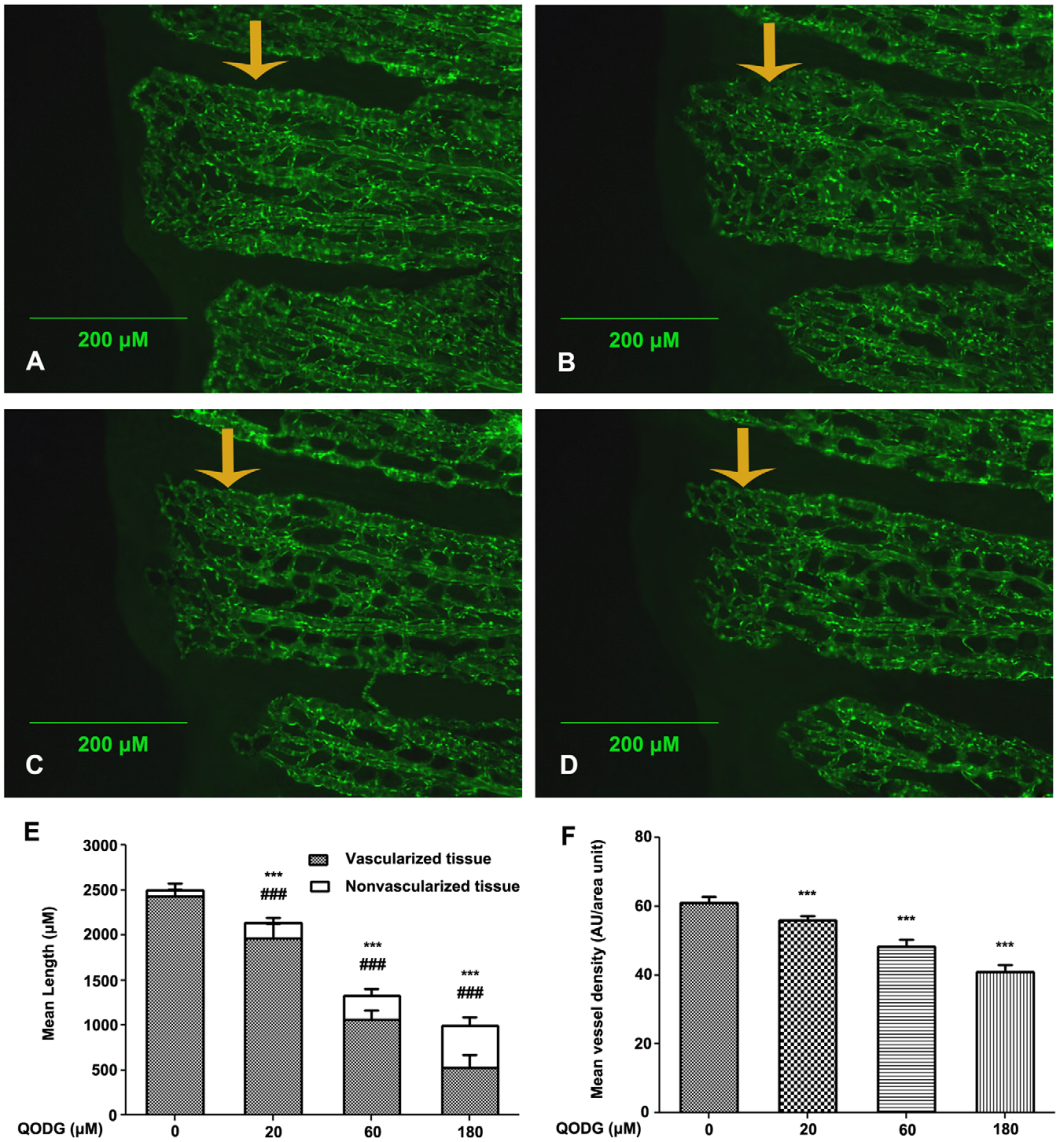
QODG inhibits angiogenesis in regenerative caudal fin of zebrafish at 7 days post amputation. (A) Vehicle control: *Tg(fli1:EGFP)* zebrafish caudal fins were clipped at the mid-fin level, then the fish were allowed to recover in 0.1% DMSO for 7 days post amputation (dpa). (B–D) QODG-treated groups: *Tg(fli1:EGFP)* zebrafish were allowed to recover in various concentrations of QODG (20, 60, 180 µM) for 7 dpa. Orange arrow indicates the amputation site in caudal fin of adult zebrafish for all figures. (E) Quantitative comparison of the lengths of regenerative vessel and fin in the vehicle control group and QODG-treated groups. Data are expressed as mean ± SD from three independent experiments. *, the length of regenerative vascularized fin tissue in QODG-treated group compared with that in the vehicle control group; ***, P<0.001 vs. vehicle control. ^#^, the length of regenerative nonvascularized fin tissue in QODG-treated group compared with that in the vehicle control group; ^###^, P<0.001 vs. vehicle control. (F) Quantitative comparison of the vessel densities of regenerative caudal fin in the vehicle control group and QODG-treated groups. Data are expressed as mean ± SD from three independent experiments. *, the vessel density of regenerative caudal fin in QODG-treated group compared with that in the vehicle control group; ***, P<0.001 vs. vehicle control. Scale bars, 200 µm.

Previous study has demonstrated that in zebrafish caudal fin, regenerative angiogenesis can be separated from fin tissue regrowth, as a small amount of avascular fin tissue, up to ∼1 mm, can be regenerated in the absence of accompanying blood vessels. However, to regenerate beyond this size limit, angiogenesis is required [53]. In order to evaluate how QODG plays a role in angiogenesis during caudal fin regeneration, we have surveyed vascularized fin tissue and nonvascularized fin tissue in regenerative caudal fin of zebrafish at 7 days post amputation (dpa). Fig. 4A showed that the lengths of regenerative caudal fin in the vehicle control group treated with 0.1% DMSO for 7 dpa were considerable, particularly, the lengths of regenerative vascularized fin tissue were largest. Following QODG treatment (20, 60, 180 µM) for 7 dpa, the lengths of regenerative vascularized fin tissue decreased obviously compared with the vehicle control group (Fig. 4B–D), with a notable dose-dependent effect indicated by quantitative analysis (Fig. 4E, P<0.001).

In the meantime, we measured the distribution of vasculature in zebrafish caudal fin at 7 days post amputation (dpa). Fig. 4A showed that the vessel densities of regenerative caudal fin in the vehicle control group treated with 0.1% DMSO for 7 dpa were most intensive by measuring the fluorescence intensity of blood vessels in regenerative caudal fin. Following QODG treatment (20, 60, 180 µM) for 7 dpa, the vessel densities of regenerative caudal fin reduced markedly compared with the vehicle control group (Fig. 4B–D), with a striking dose-dependent effect indicated by quantitative analysis (P<0.001). These data confirm that QODG inhibits angiogenesis in adult zebrafish.

### QODG inhibits cell viability in endothelial cells

**Figure 5:**
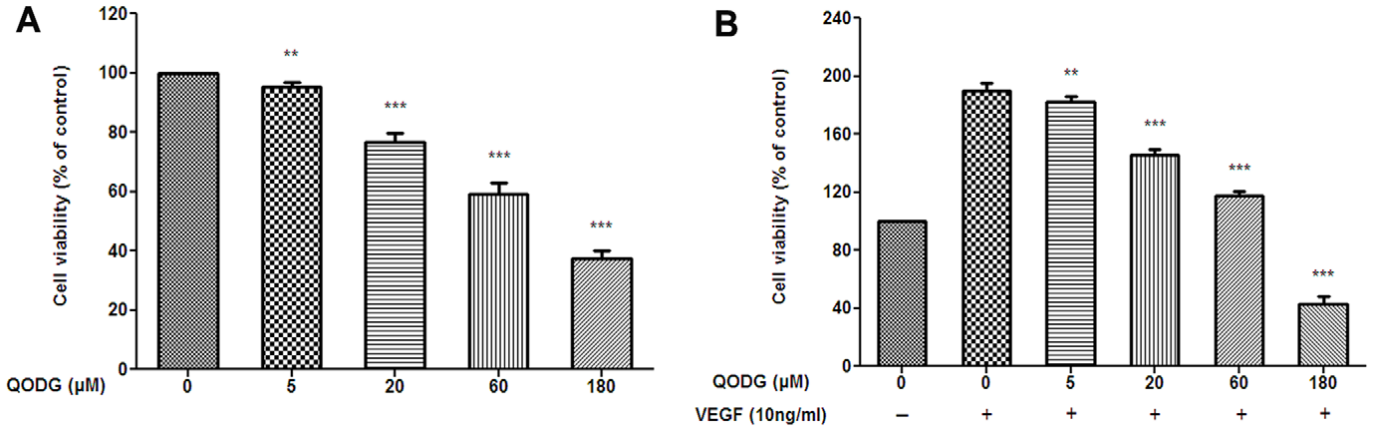
QODG inhibits cell viability in endothelial cells. (A) QODG inhibited cell viability in a dose-dependent manner under normal culture condition. HUVECs were cultured in ECGM containing 20% FBS, then cells (2×10^4^ cells/well) were treated with DMSO (0.1%) or various concentrations of QODG (5, 20, 60, 180 µM) for 24 h. Cell viability was quantified by MTS assay. Cells receiving only DMSO (0.1%) served as a vehicle control. Data are expressed as percentages of the vehicle control (100%) in mean ± SD from three independent experiments in triplicates. **, P<0.01 vs. vehicle control; ***, P<0.001 vs. vehicle control. (B) QODG inhibited cell viability in a dose-dependent manner under VEGF-induced condition. HUVECs (2×10^4^ cells/well) were starved with ECGM supplemented with 0.5% FBS for 24 h, and then treated with or without VEGF (10 ng/mL) and DMSO (0.1%) or various concentrations of QODG (5, 20, 60, 180 µM) for another 24 h. Cell viability was quantified by MTS assay. Cells receiving only DMSO (0.1%) served as a vehicle control. Data are expressed as percentages of the vehicle control (100%) in mean ± SD from three independent experiments in triplicates. **, P<0.01 vs. VEGF-treated control; ***, P<0.001 vs. VEGF-treated control.

To further elucidate the anti-angiogenic effects of QODG *in vitro*, we used MTS assays to examine human umbilical vein endothelial cell (HUVEC) proliferation and survival. Because angiogenesis is primarily initiated by growth factors, we tested whether QODG decreased VEGF-mediated HUVEC proliferation and viability. We found that when cells were cultured in normal cell culture medium (ECGM supplemented with 20% FBS) without the stimulation of VEGF, 5–180 µM of QODG observably inhibited VEGF-independent HUVEC proliferation (Fig. 5A, P<0.01 and P<0.001). Moreover, 5–180 µM of QODG significantly inhibited VEGF-mediated HUVEC survival, when cells were starved with ECGM containing 0.5% FBS for 24 h firstly, followed by the addition of DMSO (0.1%) or various concentrations of QODG (5, 20, 60, 180 µM), then stimulated with VEGF (10 ng/mL), and incubated for another 24 h (Fig. 5B, P<0.01 and P<0.001).

### QODG inhibits VEGF-induced chemotactic motility and capillary structure formation of HUVECs

**Figure 6:**
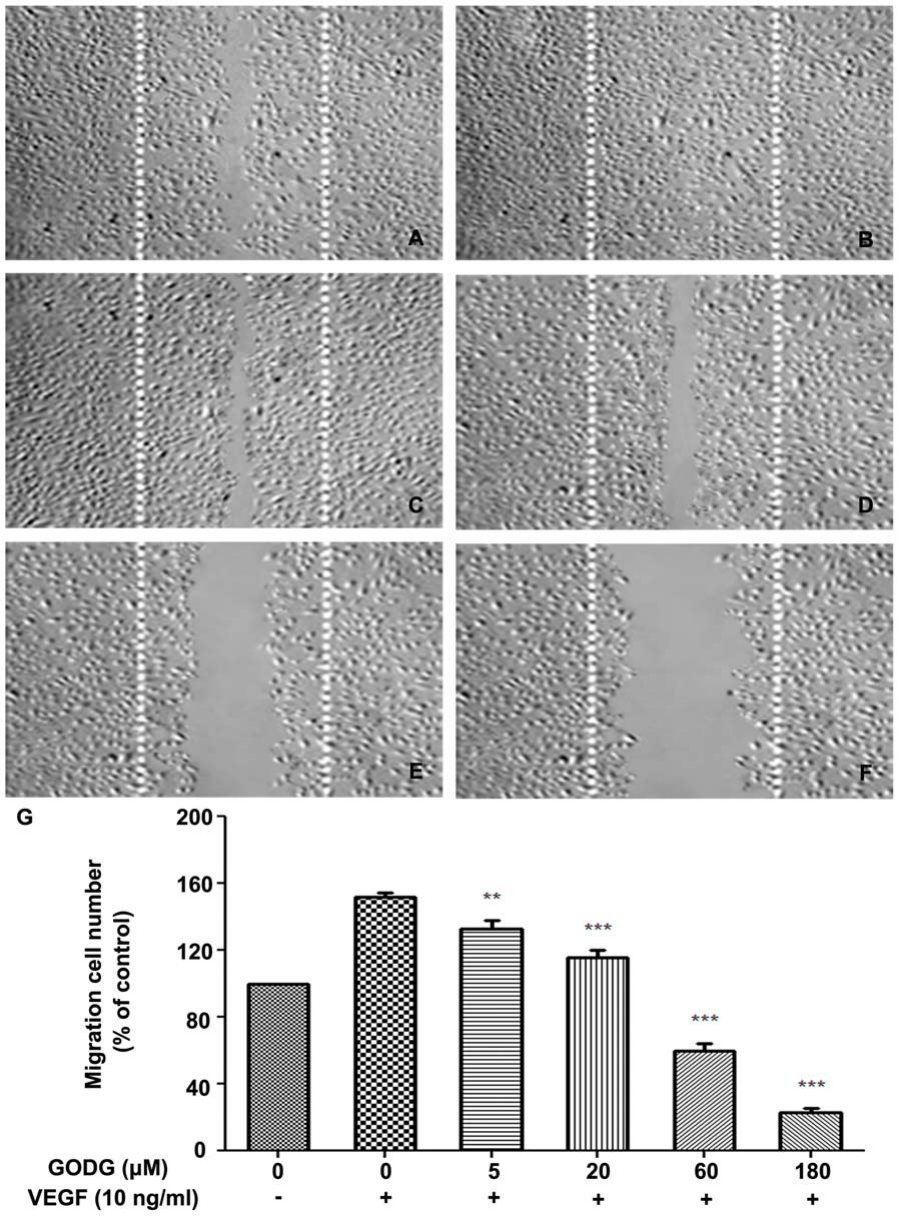
QODG inhibits VEGF–induced chemotactic motility of endothelial cells. QODG inhibited the migration of HUVECs. HUVECs were allowed to grow to full confluence in 6-well plates pre-coated with 0.1% gelatin and then starved with ECGM containing 0.5% FBS to inactivate cell proliferation. After that, cells were wounded with pipette and washed with PBS, then treated with or without VEGF (10 ng/mL) and DMSO (0.1%) or different concentrations of QODG (5, 20, 60, 180 µM) in ECGM containing 0.5% FBS. Images were taken using an inverted microscope (Olympus, Center Valley, PA, USA) (at 100×magnification) after 8 h of incubation, and migrated cells were quantified by manual counting. (A) Migration assay of HUVECs treated with only DMSO (0.1%). (B) Migration assay of HUVECs treated with VEGF (10 ng/mL) and DMSO (0.1%). (C–F) Migration assay of HUVECs treated with VEGF (10 ng/mL) and various concentrations of QODG (5, 20, 60, 180 µM). (G) Quantitative comparison of the numbers of migrated cells in different groups. Cells receiving only DMSO (0.1%) served as a vehicle control. Data are expressed as percentages of the vehicle control (100%) in mean ± SD from three independent experiments. **, P<0.01 vs. VEGF-treated control; ***, P<0.001 vs. VEGF-treated control. Scale bars, 100 µm.

As cell migration is essential for angiogenesis in endothelial cell [55], after a 6 h starvation in ECGM containing 0.5% FBS, we investigated the inhibitory effects of QODG on the chemotactic motility of endothelial cells using wound-healing migration assay. We found that QODG inhibited VEGF-induced HUVEC migration in a dose-dependent manner, with significant inhibition at 5–180 µM of QODG (Fig. 6, P<0.01 and P<0.001).

**Figure 7:**
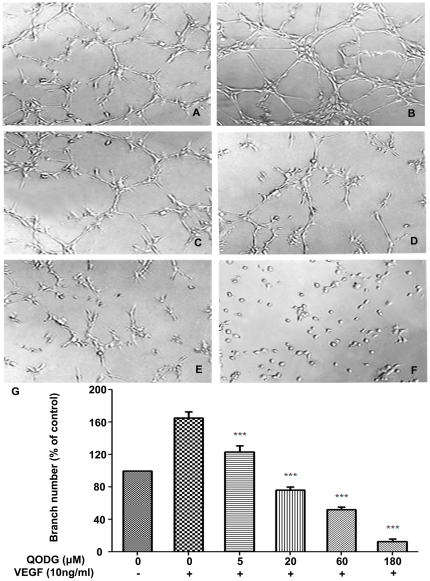
QODG inhibits VEGF-induced capillary structure formation of endothelial cells on Matrigel. QODG inhibited VEGF-induced tube formation of HUVECs. HUVECs were starved with ECGM containing 0.5% FBS, and then treated with DMSO (0.1%) or various concentrations of QODG (5, 20, 60, 180 µM). After that, cells were collected and placed in 24-well plates coated with Matrigel (4×10^4^ cells/well), followed by the activation of VEGF (10 ng/mL). After 6 h of incubation, images of the network-like structures of endothelial cells were taken using an inverted microscope (Olympus, Center Valley, PA, USA) (at 100×magnification), and branching points in different groups were quantified by manual counting. (A) HUVECs cultured on Matrigel were treated with only DMSO (0.1%). (B) HUVECs cultured on Matrigel were treated with VEGF (10 ng/mL) and DMSO (0.1%). (C–F) HUVECs cultured on Matrigel were treated with VEGF (10 ng/mL) and various concentrations of QODG (5, 20, 60, 180 µM). (G) Quantitative comparison of the numbers of branching points in different groups. Cells receiving only DMSO (0.1%) served as a vehicle control. Data are expressed as percentages of the vehicle control (100%) in mean ± SD from three independent experiments. ***, P<0.001 vs. VEGF-treated control. Scale bars, 100 µm.

Tube formation of endothelial cells is also one of the key steps of angiogenesis, although angiogenesis is a very complex procedure involving several kinds of cells [56]. To further survey the effects of QODG on endothelial cell tube formation, we used two-dimensional Matrigel assay to examine how QODG affects HUVEC tube formation. When HUVECs were seeded on the growth factor–reduced Matrigel, robust tubular-like structures were formed in the presence of VEGF. Approximately 20 µM QODG inhibited 50% VEGF-induced tube formation of HUVECs on Matrigel, and 180 µM QODG almost completely inhibited VEGF-induced tube formation of HUVECs on Matrigel (Fig. 7, P<0.001). However, it is also possible that the inhibition of vasculogenesis in matrigel at high concentrations is due to a toxic effect of QODG on cells. These results indicate that QODG may block VEGF-induced angiogenesis *in vitro* by inhibiting the migration and tubular structure formation of endothelial cells.

### QODG insignificantly changes both VEGF-induced VEGFR1 and VEGFR2 mRNAs expression in HUVECs

In order to identify molecular targets of the anti-angiogenic activity of QODG *in vitro*, mRNAs were isolated from different groups and reverse transcribed to a single-stranded cDNA, and then relative gene expression was determined using quantitative real-time PCR (qRT-PCR). VEGF is a fundamental mediator of physiological and pathological angiogenesis [57], and acts through two tyrosine kinase receptors (VEGFR2 and VEGFR1). VEGFR2 (KDR/Flk-1) has a higher affinity for VEGF and is a major transducer of the VEGF signal in endothelial cells [58], [59], whereas VEGFR1 (Flt-1) modulates VEGFR2 signaling during blood vessels formation [60]. Therefore, we examined VEGF-induced VEGFR1 and VEGFR2 mRNAs expression in HUVECs.

**Figure 8:**
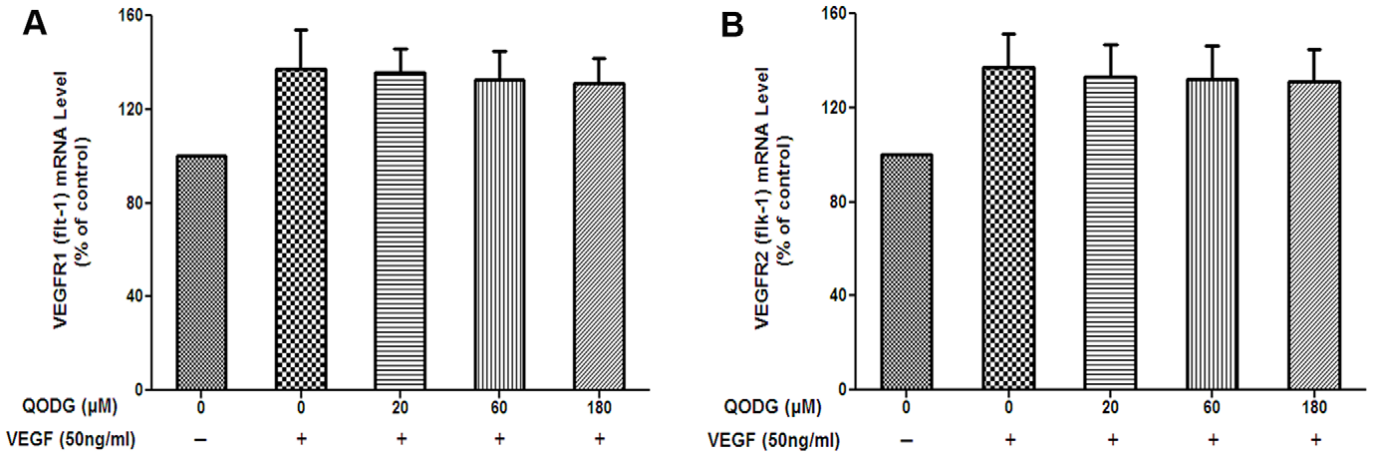
QODG insignificantly regulates the VEGF-triggered activation of VEGFR1 and VEGFR2 mRNAs expression in HUVECs. HUVECs (5×10^5^ cells/well) were treated with or without VEGF (50 ng/mL) and DMSO (0.1%) or various concentrations of QODG (20, 60, 180 µM) for 24 h. RNA was extracted with TRIzol® Reagent (Invitrogen, Life Technologies, Grand Island, NY, USA), reverse transcribed with PrimeScript™ RT reagent kit (TaKaRa, Otsu, Shiga, Japan), and quantitated by qRT-PCR using SYBR® Premix Ex Taq™ (TaKaRa, Otsu, Shiga, Japan). Cells receiving only DMSO (0.1%) served as a vehicle control. The levels of VEGFR1 and VEGFR2 mRNAs are normalized by β-actin and expressed as percentages of the vehicle control (100%) in mean ± SD from three independent experiments in triplicates. P>0.05 vs. VEGF-treated control.

The bar chart in Fig. 8 represents the genes expression in HUVECs after treatment with or without VEGF (50 ng/mL) and DMSO (0.1%) or various concentration of QODG (20, 60, 180 µM) for 24 h. There were not distinct differences in the expression of both VEGFR1 and VEGFR2 mRNAs between QODG-treated groups and the VEGF-treated control group (Fig. 8A and B, P>0.05 and P>0.05). The result suggests that the anti-angiogenic activity of QODG is not owing to down-regulation of VEGF-activated VEGFR1 and VEGFR2 mRNAs expression in HUVECs.

### QODG is a potent VEGFR2 kinase inhibitor

**Figure 9:**
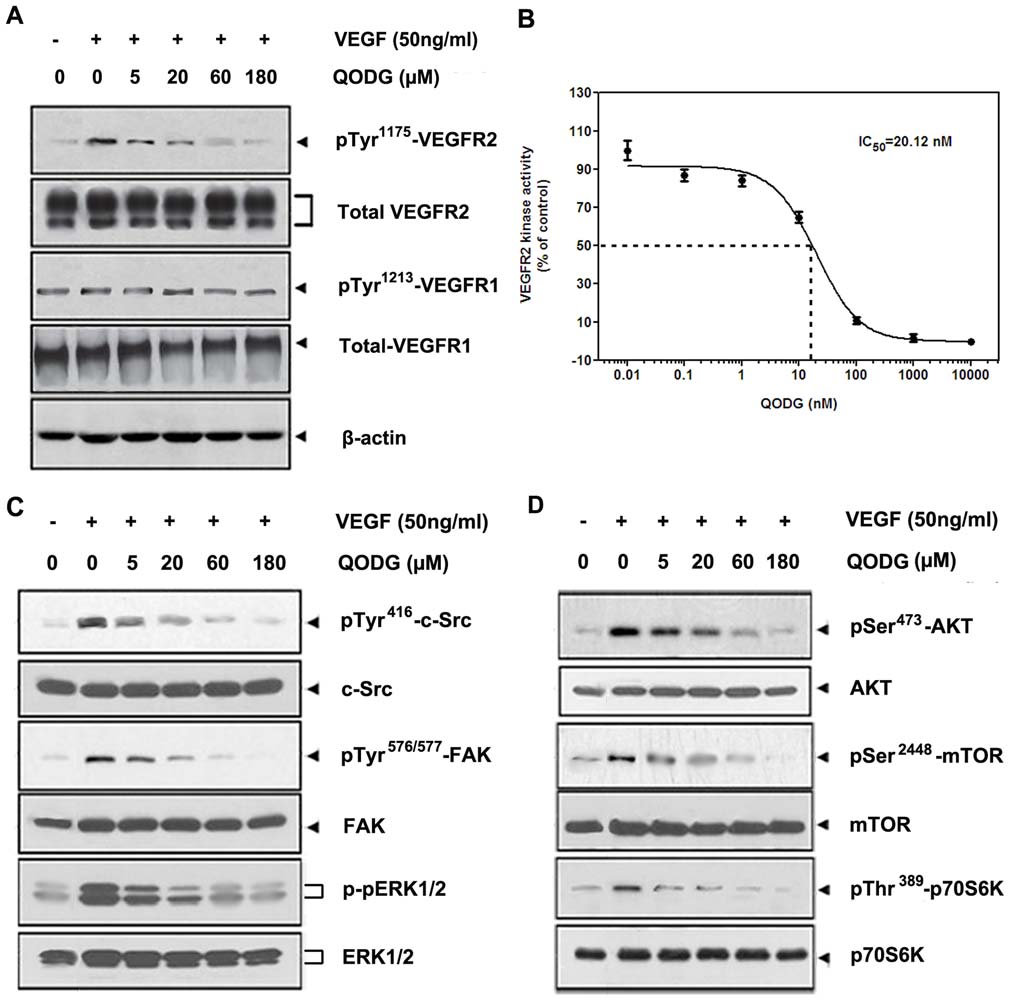
QODG inhibits VEGF-induced phosphorylation of VEGFR2 kinase and VEGFR2-mediated signaling pathway downstream molecules in HUVECs. (A) QODG inhibited VEGF-induced phosphorylation of VEGFR2 in a dose-dependent manner, but phospho-VEGFR1 protein, the total amount of VEGFR1 and VEGFR2 proteins in each sample of cells all remained comparable. After probed with the antibodies anti-VEGFR2, anti-VEGFR1, anti-phospho-VEGFR2 and anti-phospho-VEGFR1, total VEGFR2 and VEGFR1 proteins, and phospho-VEGFR2 and VEGFR1 proteins in different groups were examined by Western blotting analysis. Three independent experiments were performed in triplicates. (B) QODG inhibited VEGFR2 kinase activity. Inhibition of VEGFR2 kinase activity by QODG was analyzed using an *in vitro* HTScan® VEGF receptor 2 kinase kit (Cell Signaling Technology, Danvers, MA, USA) combined with colorimetric ELISA detection according to the manufacturer's instructions. The reaction processed with only DMSO (0.1%) served as a vehicle control. Data are expressed as percentages of the vehicle control. Three independent experiments were performed. (C, D) QODG inhibited the activation of VEGFR2-mediated downstream signaling. The activation of c-Src, FAK and ERK (C), AKT, mTOR and p70S6K (D) was suppressed by QODG. After probed with specific antibodies, proteins in different groups were examined by Western blotting analysis. Three independent experiments were performed in triplicates.

VEGFR2 is the most biologically important receptor for VEGF. It regulates endothelial cell proliferation, migration, differentiation, tube formation and angiogenesis [22]. Following its binding to VEGF, VEGFR2 dimerizes and undergoes autophosphorylation on tyrosine residues within its cytoplasmic portion [61]. A pan-phosphorylation site map of VEGFR2 has identified three major tyrosine phosphorylation site Tyr^951^, Tyr^1175^ and Tyr^1214^. In developing vessels, phosphorylation of Tyr^1175^ and Tyr^1214^ was detected in all VEGFR2-expressing endothelial cells, whereas phosphorylation of Tyr^951^ was identified in a subset of vessels [62]. Autophosphorylation of Tyr^1175^ on VEGFR2 is crucial for endothelial cell proliferation, so this region is a new target for anti-angiogenic reagents [63]. To dissect the molecular basis of QODG-mediated anti-angiogenic effects, we investigated how QODG affects VEGFR2 protein expression in HUVECs using Western blotting analysis. We found that 20 µM QODG dramatically inhibited phosphorylation of VEGFR2 Tyr^1175^, but did not inhibit total VEGFR2 protein expression (Fig. 9A).

To confirm our Western blotting analysis data and verify whether QODG directly inhibits VEGFR2 tyrosine kinase activity, we performed an ELISA-based *in vitro* VEGFR2 tyrosine kinase assay with various concentrations of QODG using HTScan® VEGFR2 kinase assay kit (Cell Signaling Technology, Massachusetts, USA) according to the manufacturer's suggested methods. As shown in Fig. 9B, QODG directly inhibited VEGFR2 tyrosine kinase activity in a dose-dependent manner with a 50% inhibitory concentration (IC_50_) of 20.12 nM. These findings suggest that QODG is a potential inhibitor of VEGFR2 tyrosine kinase.

Furthermore, VEGFR1 is described as a positive and negative regulator of VEGFR2 signalling capacity. Negative regulation is exerted, at least in part, by an alternatively spliced soluble VEGFR1 variant that binds to VEGF and thereby prevents VEGF from binding to VEGFR2 [22]. Adenoviral overexpression of endothelial soluble VEGF receptor-1 (sFlt-1) suppresses phosphorylation of VEGFR2 at Tyr^951^ and ERK1/2 MAPK, and reduces HUVEC proliferation. Consequently, VEGFR1 directly impacts on VEGFR2 signalling and acts as an autocrine regulator of endothelial cell function [64]. Recent studies have reported that VEGFR1 is autophosphorylated at Tyr^1169^, Tyr^1213^, Tyr^1242^, Tyr^1327^ and Tyr^1333^ [65]–[67], thereinto, Tyr^1213^ and Tyr^1242^ are two major phosphorylation sites. Tyr^1169^ of VEGFR1 corresponds to Tyr^1175^ of VEGFR2 which is the major site for the MAPK pathway leading towards angiogenesis [68], [69]. However, the phosphorylation of Tyr^1169^ is relatively weak [66], while Tyr^1213^ is highly autophosphorylated on VEGFR1. And interestingly, VEGFA_165_ can induce a strong phosphorylation of VEGFR1 tyrosine residue Tyr^1213^ and to lesser extent Tyr^1242^ and Tyr^1333^ [70].

Based on these facts, we examined VEGF-induced VEGFR1 phosphorylation at Tyr^1213^ in HUVECs using Western blotting analysis, and we found that QODG did not exert obvious influences on phosphorylation of VEGFR1 Tyr^1213^, as well as total VEGFR1 protein expression (Fig. 9A).

### QODG inhibits the activation of VEGFR2-mediated signaling pathways

Interaction of VEGFR2 with VEGF led to the activation of various downstream signaling molecules responsible for endothelial cell migration, proliferation and survival [71], [72]. To further delineate the mechanism that underlies the anti-angiogenic effects of QODG, we screened some key kinases involved in VEGFR2-mediated signaling pathway. We found that 20 µM QODG significantly suppressed the activation of c-Src, FAK and ERK (Fig. 9C), AKT, mTOR and p70S6K (Fig. 9D), suggesting that QODG exerted its anti-angiogenic activity through regulating the activation of VEGFR2-mediated downstream signaling cascade in endothelial cells.

### QODG potentiates apoptosis in HUVCEs

**Figure 10:**
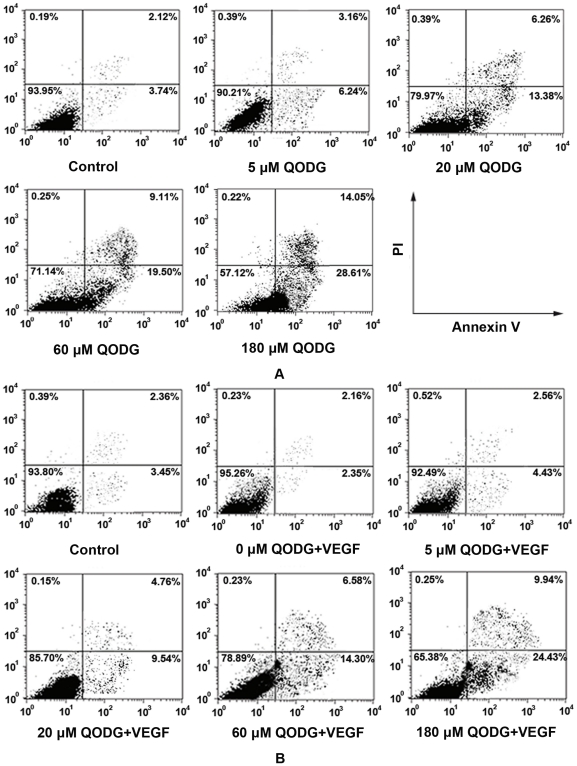
QODG induces apoptosis in HUVECs as evidenced by annexin V/PI double staining and FACS analysis. Effects of QODG on endothelial cell death were measured by annexin V-FITC/PI flow cytometry. HUVECs (2×10^6^ cells/mL) were treated with or without VEGF (10 ng/mL) and DMSO (0.1%) or various concentrations of QODG (5, 20, 60, 180 µM) for 24 h, followed by labeling for phosphatidylserine externalization with annexin V-FITC and cell membrane integrity with PI. (A) QODG induced apoptosis in HUVECs treated without VEGF in a dose-dependent manner. (B) QODG induced apoptosis in HUVECs treated with VEGF in a dose-dependent manner. The lower right quadrant (annexin-V+/PI−) represents early apoptosis, while the upper right quadrant (annexin V+/PI+) represents late apoptosis and necrosis. Data are representatives of three independent experiments with similar results.

**Figure 11:**
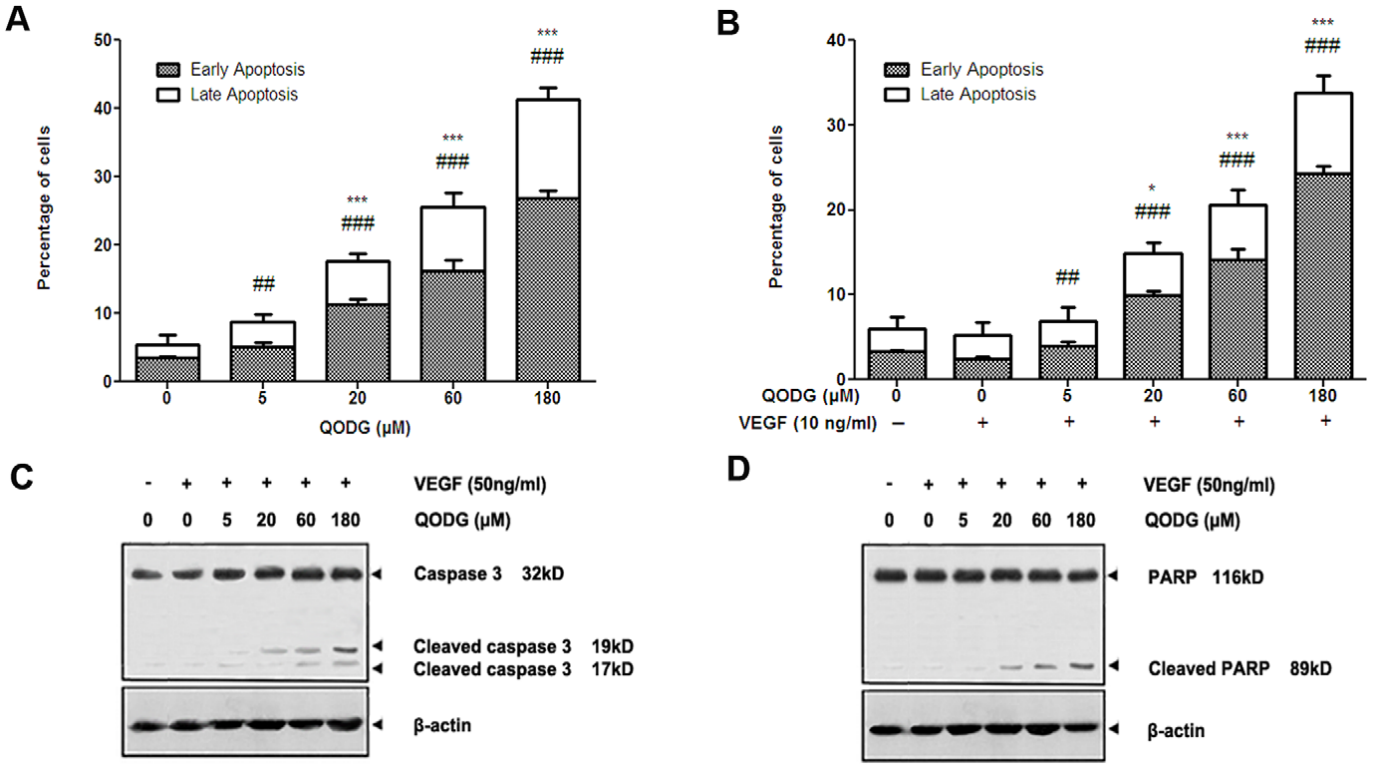
QODG potentiates apoptosis in HUVECs in a dose-dependent manner. (A–B) Relative percentages of early apoptotic cells (annexin-V+/PI−) and necrotic or late apoptotic cells (annexin-V+/PI+) were analyzed with one-way ANOVA followed by Tukey's multiple comparison test. Cells receiving only DMSO (0.1%) served as a vehicle control. Data are expressed as percentages of the vehicle control (100%) in mean ± SD from three independent experiments. (A) The percentages of early apoptotic cells and necrotic or late apoptotic cells increased in a dose-dependent manner when HUVECs were treated without VEGF. ^#^, the percentage of early apoptotic cells (annexin-V+/PI−) in QODG-treated group compared with that in the vehicle control group; ^##^, P<0.01 vs. vehicle control; ^###^, P<0.001 vs. vehicle control. *, the percentage of late apoptotic cells (annexin-V+/PI+) in QODG-treated group compared with that in the vehicle control group; ***, P<0.001 vs. vehicle control. (B) The percentages of early apoptotic cells and necrotic or late apoptotic cells increased in a dose-dependent manner when HUVECs were treated with VEGF. ^#^, the percentage of early apoptotic cells (annexin-V+/PI−) in QODG-treated group compared with that in the VEGF-treated control group; ^##^, P<0.01 vs. VEGF-treated control; ^###^, P<0.001 vs. VEGF-treated control. *, the percentage of late apoptotic cells (annexin-V+/PI+) in QODG-treated group compared with that in the VEGF-treated control group; *, P<0.05 vs. VEGF-treated control; ***, P<0.001 vs. VEGF-treated control. (C–D) QODG induced caspase-3 activation and the cleavage of PARP from its intact form to its cleaved form. Proteins from HUVECs treated with or without VEGF (10 ng/mL) and DMSO (0.1%) or various concentrations of QODG (5, 20, 60, 180 µM) were analyzed by Western blotting analysis for cleaved caspase-3 and cleaved PARP.

To clarify whether the reduction of cells viability, the inhibition of cell migration and vasculogenesis in matrigel at high concentrations of QODG is related to apoptosis, FACS analysis was carried out. HUVCEs were treated with or without VEGF (10 ng/mL) and DMSO (0.1%) or various concentrations of QODG (5, 20, 60, 180 µM) for 24 h and subjected to flow cytometry analysis after staining with annexin V-FITC and propidium iodide (PI). As shown in Fig. 10 and Fig. 11A and B, the percentages of early apoptotic cells (lower right quadrant, i.e. annexin V+/PI−) and necrotic or late apoptotic cells (upper right quadrant, i.e. annexin V+/PI+) increased in a dose-dependent manner.

To further verify whether QODG regulates endothelial cell death and confirm our FACS analysis data, we used Western blotting analysis to examine caspase-3 activation and PARP cleavage in cells treated with or without VEGF (10 ng/mL) and DMSO (0.1%) or various concentrations of QODG (5, 20, 60, 180 µM). Poly (ADP-ribose) polymerase (PARP) is a family of proteins involved in cell death [73], [74], and shown to be cleaved into 89- and 24-kD fragments that contain the active site and the DNA-binding domain of the enzyme, respectively, during drug-induced apoptosis in a variety of cells [75]–[77]. Caspase-3, a member of the caspase family of 13 aspartate-specific cysteine proteases that play a central role in the execution of the apoptotic program [78]–[80], is primarily responsible for the cleavage of PARP during cell death [76], [77], [81]. We found that addition of QODG (20–180 µM) led to the activation of caspase-3 and the cleavage of PARP from its intact form (116 kD) to its cleaved form (89 kD) markedly in endothelial cells (Fig. 11C and D), Taken together, these results suggest that QODG induces apoptosis in endothelial cells through a caspase-3-dependent pathway.

## Discussion

Angiogenesis is considered a key process in many physiological and pathological states [82], [83]. Current interest is focusing on the beneficial health effects of natural products such as flavonoids, because this kind of plant polyphenols has been found to influence some steps in cancer angiogenesis beyond their traditional use [84], [85]. Quercetin-4′-*O*-β-D-glucopyranoside (QODG) is a bioactive flavonoid from *Hypericum attenuatum* Choisy; however, little information is known about its functions in angiogenesis. In this study, we report the novel biological functions of QODG as an inhibitor of angiogenesis. Our research intensively focuses on the inhibitory effects of QODG on endothelial cell proliferation, migration and capillary structure formation as well as the stimulative effects of endothelial cell death in response to VEGF. Furthermore, we show that QODG can inhibit angiogenesis *in vivo* in zebrafish model.

Phenotypic changes of angiogensis always involve in angiogenesis-related signaling pathways. Vascular endothelial growth factor (VEGF) is a potent pro-angiogenic factor that stimulates endothelial cell proliferation, migration and tube formation, some key events of the angiogenic process [86]. The biologically relevant VEGF signaling events are mainly mediated by VEGFR2 [87]–[89]. Strong evidences are showed that blocking the activity of VEGFR2 can limit the ability of angiogenesis [87], and VEGFR inhibitors are a promising class of angiogenesis treatment drugs [88].

In the present study, we found that a half-maximum inhibitory concentration of 20.12 nM QODG significantly blocked VEGFR2 kinase activity, making QODG a potent VEGFR2 inhibitor. Our study also manifested that inhibition of VEGFR2 by QODG was not due to down-regulation of VEGF-induced VEGFR2 expression in mRNA and total protein level, but rather direct suppression of VEGF-induced phosphorylation of VEGFR2.

In clinical trials, successful anti-angiogenic therapy may require simultaneous blockade of signaling downstream from multiple pro-angiogenic factor receptors [90].

**Figure 12:**
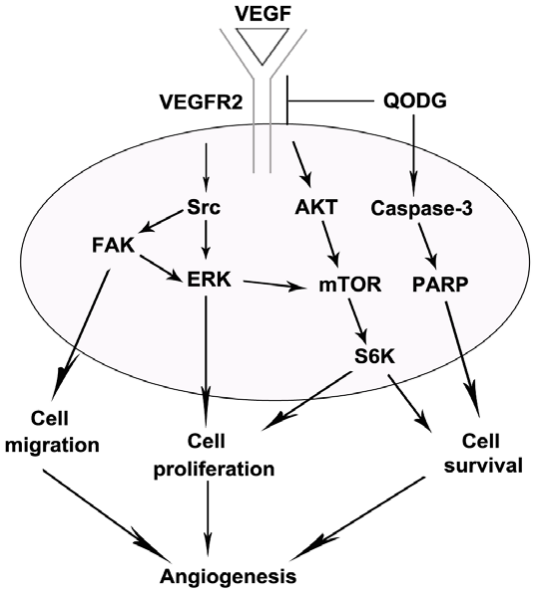
The anti-angiogenic signaling pathways regulated by QODG in HUVECs. Proposed mechanism for inhibition of angiogenesis by QODG. Arrows indicate regulations by QODG treatment in experimental results.

Previous studies have shown that the Src family kinase is substantially involved in VEGF-induced angiogenesis *in vitro* and *in vivo* [91]–[93]. By interacting between focal adhesion kinase (FAK) and Src, a dual kinase complex FAK-Src forms, and is activated by multiple integrin-regulated linkages. Activated FAK–Src functions to promote cell motility, cell cycle progression and cell survival [93]. AKT/mammalian target of rapamycin (mTOR)/ribosomal protein S6 kinase (p70S6K) signaling has also been identified as a novel, functional mediator in angiogenesis [94], [95]. In this study, we found that in endothelial cells a low concentration of QODG blocked multiple downstream signaling components of VEGFR2, c-Src (one of the Src family kinase), FAK, ERK, AKT, mTOR and p70S6K, suggesting that QODG exerted its anti-angiogenic activity through regulating the activation of VEGFR2-mediated downstream signaling cascade in endothelial cells (Fig. 12).

Interestingly, AKT/mTOR/p70S6K signaling has been found to regulate the expression of pro-angiogenic factors (e.g. VEGF) by regulating hypoxia-inducible factor-1α (HIF-1α) and interferon gamma (IFNγ) expression at the translational level [96]–[99], making the signaling a potential target for anti-angiogenic therapy.

Take VEGF for instance, VEGF is firstly described as an essential growth factor for vascular endothelial cells, and it is up-regulated in many tumors. VEGF is usually produced by numerous non-endothelial cells including tumor cells [100], [101], macrophages [102], platelets [103], keratinocytes [104], and renal mesangial cells [105]. Notably, VEGF is expressed in parenchymal cells adjacent to capillaries and vascular cells adjacent to endothelia [106], [107]. The activities of VEGF are not limited to angiogenesis, VEGF also plays a role in normal physiological functions such as bone formation [108], hematopoiesis [109], wound healing [110] and development [111]. In addition, VEGF may promote tumor growth by direct pro-survival effects in tumor cells [112], [113]. There are two major putative pathways involving in VEGF that contribute to stimulation of the neovascularization in tumor tissues. First, when tumor exhibits a rapid growth and enlargement in size, endothelial cells occurring at the hypoxic core of the tissue are known to up-regulate VEGF and stimulate themselves to be proliferated [114], [115], serving as an autocrine mechanism. Second, tumor cells enhance the VEGF generation in response to hypoxia and can thereby trigger the angiogenic responses in the adjacent endothelial cells [116], that is to say, a paracrine interaction. Therefore, QODG may suppress angiogenesis by guiding numerous non-endothelial cells such as tumor cells to produce fewer pro-angiogenic molecules (e.g. VEGF), thereby restrain angiogenesis in the adjacent endothelial cells in a paracrine fashion. Certainly, we also do not exclude the minor possibility that QODG may inhibit angiogenesis by inducing endothelial cells to produce fewer pro-angiogenic molecules in an autocrine approach.

Altogether, our study elucidates the mechanism of the anti-angiogenic activity of QODG at least in part. We have proved that QODG inhibits angiogenesis through restraining phosphorylation of VEGFR2 tyrosine kinase, suppressing VEGFR2-mediated signaling pathway which plays multiple roles in regulating neovascularization and inducing apoptosis in endothelial cells. Hence, our findings provide inspiration for further development of *Hypericum attenuatum* Choisy and Quercetin-4′-*O*-β-D-glucopyranoside (QODG) as a novel VEGFR2 kinase inhibitor for the treatment of angiogenesis-related diseases.

## Materials and Methods

### Ethics Statement

All animal experiments were conducted according to the ethical guidelines of the Institutional Animal Care and Use Committee (IACUC) of Wenzhou Medical College, and the protocol was approved by IACUC of Wenzhou Medical College. Wenzhou Medical College is accredited by the National Association for Assessment and Accreditation of Laboratory Animal Care (Approval documents: 2010/IACUC/0156).

### Chemicals and reagents

Quercetin-4′-*O*-β-D-glucopyranoside (QODG) (≥99%) was extracted from *Hypericum attenuatum* Choisy at our laboratory. QODG was dissolved in DMSO to form a 100 mM solution, stored at −20°C in small aliquots until needed and protected from light, and then diluted to various concentrations as needed. Recombinant human VEGF (VEGF_165_) was purchased from R&D Systems (Minneapolis, MN, USA). Bovine endothelial cell growth factor (bECGF) and complete proteinase inhibitor cocktail tablets were ordered from Roche (Mannheim, Baden-Württemberg, Germany). Phosphate-buffered saline (PBS), Tween 20, fetal bovine serum (FBS), bovine serum albumin (BSA), phenylmethanesulfonyl fluoride (PMSF), ethylenediaminetetraacetic acid (EDTA), heparin, HEPES buffer, penicillin, streptomycin, NaHCO_3_, amphotericin B, dimethyl sulfoxide (DMSO), gelatin and MS-222 (tricaine methanesulfonate) were obtained from Sigma (St. Louis, MO, USA). RIPA buffer, the antibodies anti-β-actin, anti-VEGFR2, anti-c-Src, anti-FAK, anti-ERK1/2, anti-AKT, anti-mTOR, anti-p70S6K, anti-caspase-3, anti-PARP, phospho-specific anti-VEGFR2 (Tyr^1175^), anti-c-Src (Tyr^416^), anti-FAK (Tyr^576/577^), anti-ERK1/2 (Thr^202^/Tyr^204^), anti-AKT (Ser^473^), anti-mTOR (Ser^2448^), anti-p70S6K (Thr^389^), anti-cleaved caspase-3 (Asp^175^), anti-cleaved PARP (Asp^214^), anti-mouse IgG HRP-linked antibody, Phototope® HRP Western blotting detection System (LumiGLO® chemiluminescent reagent and peroxide), TMB substrate and stop solution were delivered from Cell Signaling Technology (Danvers, MA, USA). The antibody anti-VEGFR1 was sent from Santa Cruz Biotechnology (Santa Cruz, CA, USA), and the antibody anti-phospho-VEGFR1 (Tyr^1213^) was dispatched from Millipore (Billerica, MA, USA). M199 medium, TRIzol® reagent and sodium dodecyl sulfate polyacrylamide electrophoresis (SDS-PAGE) gels were acquired from Invitrogen (Life Technologies, Grand Island, NY, USA).

### Embryo collection, drug treatment and measurement of the toxic effect of QODG on zebrafish

*Tg(fli1:EGFP)* transgenic zebrafish embryos were generated by natural pair-wise mating and were raised at 28.5°C in embyro water. QODG was diluted in DMSO as needed, and then was transferred to embryo water. Healthy, hatched zebrafish embryos were picked out at 6 hpf, and treated with DMSO (0.1%) or different concentrations of QODG (100, 200, 300, 400, 500, 600, 700, 800, 900, 1000 µM), then incubated in 6-well plates (10–15 embryos/well) at 28.5°C from 6 hpf to 72 hpf. Embryos receiving DMSO (0.1%) served as a vehicle control. During the period, the fish were observed for survival and morphology under an inverted microscope (Nikon, Japan) (at both 10×magnification and 100×magnification). Data were analyzed by statistical package SPSS 17.0 (SPSS Inc., Chicago, IL, USA) for non-linear regression, and LC_0_ (i.e., LC_min_) was defined as the minimum lethal concentration that resulted in 0% mortality of zebrafish treated with QODG. The assay was repeated three times independently with 30 embryos per group.

### Assessment of zebrafish embryos vascular changes and microscopy

*Tg(fli1:EGFP)* transgenic zebrafish embryos were treated with DMSO (0.1%) or various concentrations of QODG (20, 60, 180 µM), and incubated in 6-well plates (10–15 embryos/well) at 28.5°C from 6 hpf to 72 hpf. At 72 hpf, zebrafish were removed from 6-well plates, and were anesthetized with a standard solution of 0.02% MS-222 for 5–10 sec, until the tail fins stopped moving. Then the fish were transferred to slides, and observed for viability and morphological changes of vessels under a fluorescence inverted microscope (Nikon, Japan) (at 200×magnification). Images were taken with NIS-Elements F 2.30 (Nikon, Japan). The section of zebrafish just below yolk sac was chosen for the measurement of the number of complete intersegmental vessels (ISVs) and angiogenic sprouts by manual counting. Embryos receiving DMSO (0.1%) served as a vehicle control. The assay was repeated three times independently with 30 embryos per group.

### Maintenance of zebrafish and drug treatments

*Tg(fli1:EGFP)* transgenic zebrafish (*Danio rerio*) were normally maintained at 28.5°C on a 14 h light/10 h dark cycle in a recirculating tank system. QODG was diluted in DMSO as needed, then transferred to fish water. The adult zebrafish was anesthetized with a standard solution of 0.02% MS-222 for 2–4 min, until the gills stopped moving [53]. The fish were transferred to slides, and their caudal fins were promptly amputated at the mid-fin level. Then the fish were immediately placed back to a recovery tank, and nearly recovered within 2–3 min, followed by the recovery in DMSO (0.1%) or various concentrations of QODG (20, 60, 180 µM) at 28.5°C for 7 days post amputation (dpa). Up to six fish per liter were placed in a tank at one time. Zebrafish receiving DMSO (0.1%) served as a vehicle control. The assay was repeated three times independently with 10 fins per group.

### Fin vascularization measurements and microscopy

At 7 dpa, the adult zebrafish were anesthetized with a standard solution of 0.02% MS-222 for 2–4 min, until the gills stopped moving [53]. Then the fish were transferred to slides, and the measurements of caudal fin regeneration were carried out only when each fin ray was responding consistently to treatment. As the fin rays curved toward the midline, rays 2, 3, 4 and 5 from the edges of the caudal fin provide the most consistent growth and vascularization [53]. Thereby the fish were placed under a fluorescent inverted microscope (Nikon, Japan) (at 200×magnification) to assess functional blood vessels in the second ray from the dorsal edge of the fin, then immediately placed back to a recovery tank, and nearly recovered within 2–3 min. The images were taken of adult tail fins at the mid-fin level with NIS-Elements F 2.30 (Nikon, Japan). The length of regenerative caudal fin was measured by image analysis software Image-Pro Plus 6.0 (Silver Spring, MD, USA). The vessel density of caudal fin was indirectly estimated through the fluorescence intensity of blood vessels in regenerative caudal fin by another image analysis software NIS-Elements BR 2.30 (Nikon, Japan).

### Cell lines and cell culture

Human umbilical vascular endothelial cells (HUVECs) (Clonetics, Lonza, Basel, Switzerland) were cultured in endothelial cell growth medium (ECGM): M199 medium supplemented with 20% FBS, 20 µM bECGF, 0.1 mg/mL heparin, 15 mM HEPES buffer, 50 IU/L penicillin, 50 mg/L streptomycin, 44 mM NaHCO_3_, and 50 µg/mL amphotericin B at 37°C under a humidified 95%∶5% (v/v) mixture of air and CO_2_ [117].

### Cell viability assay

Firstly, examine the effects of QODG on HUVEC viability under normal culture condition. HUVECs were cultured in ECGM containing 20% FBS. When grew to 2×10^4^ cells/well, endothelial cells were treated with DMSO (0.1%) or various concentrations of QODG (5, 20, 60, 180 µM) for 24 h. Cell viability was determined by MTS assay following the manual of CellTiter 96® AQ_ueous_ One Solution Cell Proliferation Assay (Promega, Madison, WI, USA) with VersaMax™ microplate reader (Molecular Devices, Sunnyvale, CA, USA) at 490 nm. Three independent experiments were performed in triplicates.

Secondly, examine the effects of QODG on HUVEC viability under VEGF-induced condition. HUVECs (2×10^4^ cells/well) were starved with ECGM containing 0.5% FBS for 24 h. After the pre-incubation, cells were treated with or without VEGF (10 ng/mL) and DMSO (0.1%) or various concentrations of QODG (5, 20, 60, 180 µM), and incubated for another 24 h. Cell viability was also quantified by MTS assay. Three independent experiments were performed in triplicates.

Cells receiving only DMSO (0.1%) served as a vehicle control. Inhibition percentage was expressed as percentage of the vehicle control (100%).

### Endothelial cell migration assay

HUVECs were allowed to grow to full confluence in 6-well plates pre-coated with 0.1% gelatin and then starved with ECGM containing 0.5% FBS for 6 h to inactivate cell proliferation. After that, cells were wounded with pipette tips and washed with PBS. ECGM supplemented with 0.5% FBS was added into the wells with or without VEGF (10 ng/mL) and DMSO (0.1%) or various concentration of QODG (5, 20, 60, 180 µM). Images of cells were taken using an inverted microscope (Olympus, Center Valley, PA, USA) (at 100×magnification) after 8 h of incubation at 37°C in a 95%∶5% (v/v) mixture of air and CO_2_. The migrated cells were observed from three randomly selected fields and quantified by manual counting. Cells receiving only DMSO (0.1%) served as a vehicle control. Inhibition percentage was expressed as percentage of the vehicle control (100%). The assay was repeated three times independently

### Endothelial cell capillary-like tube formation assay

Matrigel™ Basement Membrance Matrix (growth factor reduced) (BD Biosciences, San Jose, CA, USA) was thawed at 4°C, pipetted into prechilled 24-well plates (100 µL Matrigel/well), and incubated at 37°C for 45 min. HUVECs were firstly incubated in ECGM supplemented with 0.5% FBS for 6 h and then treated with DMSO (0.1%) or various concentrations of QODG (5, 20, 60, 180 µM) for 30 min before seeding. Cells were collected and placed onto the layer of Matrigel (4×10^4^ cells/well) in 1 mL of ECGM supplemented with 0.5% FBS, followed by the addition of VEGF (10 ng/mL). After 6 h of incubation at 37°C in a 95%∶5% (v/v) mixture of air and CO_2_, the network-like structures of endothelial cells were examined under an inverted microscope (Olympus, Center Valley, PA, USA) (at 100×magnification). The tube-like structures were defined as endothelial cord formations that were connected at both ends [118]. Branching points in three random fields per well was quantified by manual counting. Cells receiving only DMSO (0.1%) served as a vehicle control. Inhibition percentage was expressed as percentage of the vehicle control (100%). The assay was repeated three times independently.

### FACS analysis

In order to determine whether the reduction of cells viability, the inhibition of cell migration and vasculogenesis in matrigel at high concentrations of QODG is related to apoptosis, FACS analysis was carried out in the absence of VEGF or stimulated by VEGF. In brief, HUVECs (2×10^6^ cells/mL) were treated with or without VEGF (10 ng/mL) and DMSO (0.1%) or various concentrations of QODG (5, 20, 60, 180 µM) for 24 h. Then cells were harvested by trypsinization, washed twice with PBS, and resuspended in binding buffer. Both detached and adherent cells were collected and stained with annexin V-FITC and propidium iodide (PI) according to the manufacturer's instruction (BD Biosciences Pharmingen, San Diego, CA, USA). Then the stained cells were immediately evaluated immediately by flow cytometry on a FACScalibur™ system (BD Biosciences, Franklin Lakes, NJ, USA) followed by analysis using CellQuest™ Pro software (BD Biosciences, Franklin Lakes, NJ, USA).

### Total RNA extraction, reverse transcription and quantitative real-time PCR

The effects of QODG on certain genes were determined by quantitative real-time PCR (qRT-PCR). HUVECs (5×10^5^ cells/well) were seeded in 24-well plates, and starved with ECGM containing 0.5% FBS for 24 h. After the pre-incubation, cells were treated with or without VEGF (50 ng/mL) and DMSO (0.1%) or various concentrations of QODG (20, 60, 180 µM) for another 24 h. Then cells were harvested in TRIzol® reagent, and their RNA was extracted, reconstituted in DEPC-treated water, and checked for integrity by agarose-gel electrophoresis. RNA samples were quantified at OD_260/280_, and RNA was introduced to reverse transcribe to single-stranded cDNA using PrimeScript™ RT reagent kit (TaKaRa, Otsu, Shiga, Japan), followed by qTR-PCR using the SYBR® Premix Ex Taq™ (TaKaRa, Otsu, Shiga, Japan) in Mastercycler® ep gradient realplex2 Real-Time PCR System (Eppendorf, Hamburg, Germany).

The reverse-transcribed RNA was primed with oligonucleotides specific for VEGFR1 (Flt-1) (forward: 5′–GGGCAGACTCTTGTCCTCAACT–3′ and reverse: 5′–CAGCTCATTTGCACCCTCGT–3′), VEGFR2 (Flk-1) (forward: 5′–GACTGTGGCGAAGTGTTTTTGA–3′ and reverse: 5′–GTGCAGGGGAGGGTTGGCGTAG–3′), and β-actin (forward: 5′–GTGCGGGACATCAAGGAGAA–3′ and reverse: 5′–AGGAAGGAGGGCTGGAAGAG–3′) (Applied Biosystems, Carlsbad, CA, USA).

The PCR program was set as below: 94°C 3 min, (94°C 30 s, 55°C 30 s, 72°C 1 min, read plate)×30 cycles; 72°C 5 min. A standard curve for each gene was generated to monitor amplification efficiency and to relatively quantify mRNA abundance. mRNA abundance was normalized to β-actin levels and expressed as percentage of the vehicle control (100%) for statistical analysis. Cells receiving only DMSO (0.1%) served as a vehicle control. Three independent experiments were performed in triplicates.

### Western blotting analysis

To determine the effects of QODG on VEGFR2-mediated signaling cascade, HUVECs were firstly starved in ECGM containing 0.5% FBS for 12 h. After being washed with fresh medium, cells were treated with DMSO (0.1%) or various concentrations of QODG (5, 20, 60, 180 µM) for 30 min, followed by the stimulation with 50 ng/mL of VEGF for 2 min (for VEGFR2 phosphorylation) or 20 min (for AKT/mTOR/p70S6K pathway phosphorylation, ERK pathway phosphorylation and caspase-3 pathway phosphorylation). The whole-cell extracts were prepared in RIPA buffer supplemented with PMSF and proteinase inhibitor cocktail before use. Proteins are resolved by electrophoresis then transferred out of the SDS-PAGE gel and onto polyvinylidene difluoride (PVDF) membranes (Schleicher and Schuell BioScience, Keene, NH, USA). The membranes were incubated with primary antibodies anti-β-actin, anti-VEGFR2, anti-VEGFR1, anti-c-Src, anti-FAK, anti-ERK1/2, anti-AKT, anti-mTOR, anti-p70S6K, anti-caspase-3, anti-PARP, phospho-specific anti-VEGFR2 (Tyr^1175^), anti-VEGFR1 (Tyr^1213^), anti-c-Src (Tyr^416^), anti-FAK (Tyr^576/577^), anti-ERK1/2 (Thr^202^/Tyr^204^), anti-AKT (Ser^473^), anti-mTOR (Ser^2448^) and anti-p70S6K (Thr^389^), anti-cleaved caspase-3 (Asp^175^), anti-cleaved PARP (Asp^214^) followed by the addition of secondary (anti-mouse) antibodies conjugated to horseradish peroxidase (HRP). Proteins bands were visualized using Phototope® HRP Western blotting detection System (LumiGLO® chemiluminescent reagent and peroxide) according to the manufacturer's protocol. Cells receiving only DMSO (0.1%) served as a vehicle control. Three independent experiments were performed in triplicates.

### *In vitro* VEGFR2 kinase inhibition assay

*In vitro* the ability of QODG to inhibit VEGFR2 tyrosine kinase activity was assayed by prediluted QODG following the manual of HTScan® VEGFR2 kinase assay kit (Cell Signaling Technology, Danvers, MA, USA). 4×reaction cocktail containing VEGFR2 was incubated with prediluted QODG or DMSO (0.1%) for 5 min at room temperature, and then 2×ATP/substrate peptide cocktail was added to the pre-incubated reaction cocktail/QODG compound or DMSO (0.1%). After incubation at room temperature for 30 min, stop the reaction by stop buffer. Then each reaction was transferred to a 96-well streptavidin-coated plate (PerkinElmer Life Sciences, Boston, MA, USA), and incubated for 1 h at room temperature. Primary antibody [phosphorylated tyrosine monoclonal antibody (pTyr-100), 1∶1000 in PBS/T with 1% bovine serum albumin (BSA)] was added into per well until the wells were washed thrice with PBS/T (1×PBS, 0.05% Tween-20). After incubated at room temperature for 1 h, phosphorylation of the substrate was monitored with HRP-labeled anti-mouse IgG antibody (1∶500 in PBS/T with 1% BSA), followed by a chromogenic reaction. Finally, the VEGFR2 kinase assay was detected at 450 nm with VersaMAX™ microplate reader (Molecular Devices, Sunnyvale, CA, USA). The reaction processed with only DMSO (0.1%) served as a vehicle control. The results were expressed as percents kinase activity of the vehicle control (100%), and IC_50_ was defined as the compound concentration that resulted in 50% inhibition of enzyme activity. The kinase assay was performed thrice independently.

### Statistical analysis

Data were analyzed with one-way ANOVA followed by Tukey's multiple comparison test using GraphPad Prism 5.0 (GraphPad Software, San Diego, CA, USA). Non-linear regression was performed by statistical package SPSS 17.0 (SPSS Inc., Chicago, IL, USA). Curve fitting was carried out using GraphPad Prism 5.0 (nonlinear fit, variable slope sigmoidal dose-response model). Data were expressed as mean ± SD from at least three independent experiments. Differences were considered as significant at P<0.05. Livak (2^−ΔΔCt^) mathematical model, i.e., ratio = 2^−[Ct(target,test)−Ct(target,calibrator)]−[Ct(ref,test)−Ct(ref,calibrator)]^ is used for quantitative PCR analysis.
